# Feasibility of predicting maximal oxygen uptake by using the efficiency factor in healthy men

**DOI:** 10.1038/s41598-023-43307-3

**Published:** 2023-10-05

**Authors:** Fang Li, Yu-Tsai Tu, Hung-Chih Yeh, Chia-An Ho, Cheng-Pang Yang, Ying-Chen Kuo, Chin-Shan Ho

**Affiliations:** 1 School of Physical Education, Central China Normal University, Wuhan, People’s Republic of China; 2 Postdoctoral Research Mobile Station of Physical Education, Central China Normal University, Wuhan, People’s Republic of China; 3 Graduate Institute of Sports Science, Guishan District, National Taiwan Sport University, No. 250, Wenhua 1st Rd., Taoyuan City, Taiwan; 4 Department of Physical Medicine and Rehabilitation, Taipei City Hospital, Zhengzhou Branch, Taipei City, Taiwan; 5 Department of Orthopedic Surgery, Division of Sports Medicine Chang Gung Memorial Hospital, College of Medicine, Chang Gung University, Linkou, Taiwan; 6 Department of Physical Medicine and Rehabilitation, Shin Kong Wu Ho-Su Memorial Hospital, Taipei, Taiwan

**Keywords:** Physiology, Health care

## Abstract

Conventionally, efficiency is indirectly estimated through a respiratory gas analyser (oxygen, carbon dioxide), which is a complex and rather costly calculation method that is difficult to perform in many situations. Therefore, the present study proposed a modified definition of efficiency, called the efficiency factor (EF) (i.e., the ratio of work to the corresponding exercise intensity), and evaluated the relation between the EF and maximal oxygen uptake (${\dot{\text{V}}\text{O}}_{2\max }$), as well as compared the prediction models established based on the EF. The heart rate (maximal heart rate: 186 ± 6 beats min^−1^), rating of perceived exertion (19 ± 1), and ${\dot{\text{V}}\text{O}}_{2\max }$ (39.0 ± 7.1 mL kg^−1^ min^−1^) of 150 healthy men (age: 20 ± 2 years; height: 175.0 ± 6.0 cm; weight: 73.6 ± 10.7 kg; body mass index [BMI]: 24.0 ± 3.0 kg m^−2^; percent body fat [PBF]: 17.0 ± 5.7%) were measured during the cardiopulmonary exercise test (CPET). Through multiple linear regression analysis, we established the BMI model using age and BMI as parameters. Additionally, we created the PBF model^HRR^ utilizing weight, PBF, and heart rate reserve (HRR) and developed PBF model^EF6^ and PBF model^EF7^ by incorporating EF6 from the exercise stage 6 and EF7 from the exercise stage 7 during the CPET, respectively. EF6 (r = 0.32, *p* = 0.001) and EF7 (r = 0.31, *p* = 0.002) were significantly related to ${\dot{\text{V}}\text{O}}_{2\max }$. Among the models, the PBF model^EF6^ showed the highest accuracy, which could explain 62.6% of the variance in the ${\dot{\text{V}}\text{O}}_{2\max }$ at with a standard error of estimate (SEE) of 4.39 mL kg^−1^ min^−1^ (%SEE = 11.25%, *p* < 0.001). These results indicated that the EF is a significant predictor of ${\dot{\text{V}}\text{O}}_{2\max }$, and compared to the other models, the PBF model^EF6^ is the best model for estimating ${\dot{\text{V}}\text{O}}_{2\max }$.

## Introduction

Known as one of the basic elements of physical fitness, cardiorespiratory fitness refers to the capacity of the circulatory and respiratory systems to supply oxygen to the skeletal muscle mitochondria for the energy production needed during physical activity^1^. Low cardiorespiratory fitness is a strong predictor of diabetes, cardiovascular disease, hypertension, hypercholesterolemia and all-cause mortality in adults. Improving cardiorespiratory fitness can effectively reduce the risk of cardiovascular disease and all-cause mortality^1,2^. Maximal oxygen uptake (${\dot{\text{V}}\text{O}}_{2\max }$) is an important sports performance and health outcome and is a recognized indicator for assessing cardiorespiratory fitness, which has been confirmed by evidence from various studies^3–5^. Therefore, it has been deemed necessary to evaluate the cardiorespiratory fitness of adults through ${\dot{\text{V}}\text{O}}_{2\max }$ measurements.

The most direct and accurate method to measure ${\dot{\text{V}}\text{O}}_{2\max }$ is to have participants perform a cardiopulmonary exercise test (CPET) on a treadmill or cycle ergometer until exhaustion while monitoring their physical status using a gas exchange analyser. Nevertheless, this kind of direct measurement requires highly precise laboratory techniques, expensive devices and large exercise loads, which is time-consuming and requires well-trained personnel to perform the complicated operating procedures. For large-scale ${\dot{\text{V}}\text{O}}_{2\max }$ tests, the age and physical conditions of participants may vary greatly, and maximum exercise can increase the risk of adverse cardiovascular events in individuals with low cardiorespiratory fitness^6^. Furthermore, participants’ motivation may change during the maximal exercise test in response to exercise intensity and duration^7^. Similar to all maximal exercise tests, the cardiorespiratory fitness test is susceptible to the impact of motivation^1^. Insufficient participant motivation presents a challenge in accurately identifying the absence of ${\dot{\text{V}}\text{O}}_{2\max }$ achievement^8^. As these subjective and objective factors limit the direct measurement of ${\dot{\text{V}}\text{O}}_{2\max }$ in home testing and widespread applications, it is necessary to develop a low-risk, low-cost, high-efficiency and more convenient indirect ${\dot{\text{V}}\text{O}}_{2\max }$ measurement approach to evaluate the cardiorespiratory fitness of adults.

Many studies have proposed diverse submaximal exercise testing approaches^9–12^, with a considerable level of reliability and validity, and demonstrated the application value thereof in terms of cardiorespiratory fitness evaluation. The most common testing methods include the 6-min walk test^13^, the 20-m shuttle run^14^ and the step test^15,16^. These studies use exercise parameters, such as heart rate, speed and distance, as predictive factors of ${\dot{\text{V}}\text{O}}_{2\max }$ and combine them with age, sex and other physiological parameters to establish ${\dot{\text{V}}\text{O}}_{2\max }$ prediction formulas to assess cardiorespiratory fitness in adults. However, these tests are not suitable for individuals with restrictions such as overweight, pain, gait abnormalities, and those with impaired balance. Cycle ergometers are a popular exercise mode and have a lower body load requirement than running, walking and stepping. In addition, cycle ergometers are easier to use for individuals who are overweight or suffer from limitations in walking, stepping and running, as they require less sports skills and coordination. These advantages have promoted the development of submaximal cycling tests, including the Astrand-Ryhming, Young Men’s Christian Association (YMCA) and Ekblom-Bak tests^17–20^. However, it has been observed that prediction models established through these submaximal cycle ergometer tests tend to overestimate ${\dot{\text{V}}\text{O}}_{2\max }$ in healthy men with low fitness levels and underestimate it in those with high fitness levels^21,22^. Consequently, there is a pressing need to develop a new formula that can provide a more precise and individualized estimation of ${\dot{\text{V}}\text{O}}_{2\max }$ in healthy men.

Mechanical efficiency should be considered in cardiorespiratory fitness evaluation tests^23^. In physics, mechanical efficiency is a critical concept. It refers to the ratio of useful work to total work. A higher mechanical efficiency indicates a larger proportion of useful work. In physiology, mechanical efficiency represents the ratio of mechanical work (W) to energy expenditure (E)^24^. In other words, mechanical efficiency quantifies the energy consumed when performing measured external work^25^. Human mechanical efficiency is determined by workload, speed, active muscle mass, physical coordination, and individual training status^26^. When completing the same load, individuals who have been trained can have a higher mechanical efficiency due to a lower total energy expenditure. For many patient populations, high mechanical efficiency is essential in terms of using limited resources effectively and preventing mechanical overuse. As poor exercise economy can lead to invalid or inaccurate maximal cardiorespiratory fitness test results^23^, it is crucial to establish cardiorespiratory fitness evaluation methods related to efficiency in both theory and practice.

Efficiency is an essential measurement that can be used to evaluate sports performance and the effects of training and motor learning^25,26^. Nevertheless, mechanical efficiency parameters frequently used in the past were indirectly estimated through an expired respiratory gas analyser^24,26^, which involves complicated formulas that are challenging to apply in many situations, especially in clinical settings. Therefore, in this study, we proposed a modified definition of efficiency, called the efficiency factor (EF), and defined the EF as the ratio of work (W) to the corresponding exercise intensity (percentage of heart rate reserve, %HRR). Due to the uncertain relationship between the EF and ${\dot{\text{V}}\text{O}}_{2\max }$, we assumed that there would be a significant positive relation between the EF and ${\dot{\text{V}}\text{O}}_{2\max }$, where individuals with higher cardiorespiratory fitness would have a higher EF at the same workload. Furthermore, the use of EF parameters will contribute to establishing a more accurate ${\dot{\text{V}}\text{O}}_{2\max }$ prediction model. The aim of the present study was (1) to determine the relationship between the EF and ${\dot{\text{V}}\text{O}}_{2\max }$, (2) to evaluate the effectiveness of the EF as a predictor of ${\dot{\text{V}}\text{O}}_{2\max }$ and (3) to compare the predictive validity of different models in healthy male adults.

## Methods

### Study design

The participants underwent (1) anthropometric measurements and (2) an incremental test to determine ${\dot{\text{V}}\text{O}}_{2\max }$. The participants had a total of two visits, and data collection spanned a total of approximately two hours, divided into two periods on weekdays: 8:00 am to 12:00 pm and 1:00 pm to 6:00 pm. The Lode Excalibur Sport electromagnetically braked cycle (Lode BV, Groningen, the Netherlands) and the cardiopulmonary exercise testing system (CPX/ULTIMA™, MGC Diagnostics, USA) were adopted to directly measure the ${\dot{\text{V}}\text{O}}_{2\max }$ of all participants. As participants’ EFs during the CPET were significantly related to ${\dot{\text{V}}\text{O}}_{2\max }$, they were considered predictive factors in this study. This study also used the efficiency factor from stage 6 (EF6) and stage 7 (EF7) of the CPET and the variables of age, weight, HRR (220-age-resting heart rate), body mass index (BMI), and percent body fat (PBF) to establish ${\dot{\text{V}}\text{O}}_{2\max }$ prediction models. The BMI model included age and BMI. The PBF model^HRR^ included weight, HRR, and PBF. The PBF model^EF6^ incorporates weight, HRR, PBF, and EF6. The PBF model^EF7^ involves weight, HRR, PBF, and EF7. To examine the stability of these four ${\dot{\text{V}}\text{O}}_{2\max }$ prediction models, this study employed the predicted residual error sum of squares (PRESS) statistical method to cross-validate the models. The procedures of this study were approved by the Institutional Review Board of Fu Jen Catholic University (New Taipei City, Taiwan) (reference number: C108100), and all methods were performed in accordance with relevant guidelines and regulations. It conformed to the principles of the Declaration of Helsinki.

### Participants

A total of 150 healthy males without training experience (age: 20 ± 2 years; height: 175.0 ± 6.0 cm; weight: 73.6 ± 10.7 kg; BMI: 24.0 ± 3.0 kg m^−2^; PBF: 17.0 ± 5.7%; HRR: 128 ± 13 beats min^−1^) participated in this study. They underwent body composition measurements and cardiopulmonary exercise tests. Participants were instructed to maintain their regular daily routines and dietary habits throughout the study period while refraining from consuming alcoholic beverages and engaging in vigorous physical activity before the incremental test. Individuals with cardiovascular disease, hypertension, asthma, or upper limb, lower limb, or musculoskeletal injuries within the past three months were excluded. Prior to participating in this study, all participants signed informed consent forms after the content and procedures of this study were explained. This study used a body composition analyser (InBody ® 570, Biospace, Inc. Seoul, Korea) to measure participants’ weight and PBF, and their BMI was calculated by dividing their weight (kg) by the square of their height (m^2^). To ensure the accuracy of body composition measurements, participants were required to avoid eating or exercising for at least 8 h and to avoid consuming alcohol or excessive caffeine for at least 24 h prior to the test.

### Cardiopulmonary exercise test

This study used a Lode Excalibur Sport electromagnetically braked cycle with a cardiopulmonary exercise testing system to directly measure participants’ ${\dot{\text{V}}\text{O}}_{2\max }$. The cardiopulmonary exercise testing system was utilized to measure oxygen (O_2_) consumption, carbon dioxide (CO_2_) production, and pulmonary ventilation on a breath-by-breath basis. In adherence to the manufacturer's guidelines, the flow sensor and O_2_ and CO_2_ were calibrated prior to each test to ensure precise measurements. An automatic calibration feature was utilized for O_2_ and CO_2_ calibration. The automatic calibration of the gas takes signal measurements and automatically adjusts the gain and offset values to match the signal values. The electromagnetically braked cycle offers adjustable settings to ensure participant comfort and proper positioning. Before the test, we performed calibration and assisted participants in adjusting the seat height, handlebar position, and pedal placement. During the CPET, participants had to wear a chest strap heart rate sensor (Polar H10, Polar Electro Oy, Finland) to continuously monitor their heart rate response. At the same time, participants also wore a suitable respirator mask, which was connected to a sampling line and a digital flow sensor to measure oxygen uptake (${\dot{\text{V}}\text{O}}_{2}$) and the content of produced carbon dioxide (${\dot{\text{V}}\text{CO}}_{2}$). The initial load for the CPET was 25 W, with an increase of 15 W every two minutes (i.e., one stage every two minutes) until the participants could no longer maintain a pedalling frequency of 70 rpm^12,27^. At the same time, the Borg Rating of Perceived Exertion (RPE, 6–20) was used to ask participants about their level of fatigue during the CPET. In this study, participants who met any three of the following ${\dot{\text{V}}\text{O}}_{2\max }$ criteria were considered to have reached exhaustion: (1) the load increased and the oxygen uptake did not increase but slightly decreased; (2) the maximum respiratory exchange ratio was ≥ 1.10; (3) the exercise heart rate reached 90% of the age-predicted maximum heart rate (220-age); and (4) RPE score was > 17^11,12^.

### Efficiency factor

Based on the concept of mechanical efficiency, we propose a modified definition of efficiency, namely, the EF. In this study, the EF was defined as the ratio of work (W) to the corresponding exercise intensity (% HRR). The participants’ average heart rate at each stage was used to calculate the corresponding exercise intensity (%HRR). With the use of the EF formula, we calculated the participants’ EFs for the first seven consecutive exercise stages during the CPET (Stages 1 to 7) and recorded them as EF1, EF2, EF3, EF4, EF5, EF6, and EF7, respectively.

### Statistical analysis

The Shapiro‒Wilk test was adopted in this study to assess the normality of all parameters, and independent sample t tests (for normally distributed data) and Mann‒Whitney *U* tests (for nonnormally distributed data) were used to analyse differences between the derivation and validation groups in terms of anthropometric parameters, body composition, HRR, ${\dot{\text{V}}\text{O}}_{2\max }$, and EFs. The Pearson correlation coefficient was calculated to analyse the linear relationship between the measured ${\dot{\text{V}}\text{O}}_{2\max }$ and age, weight, BMI, PBF, HRR and EFs in the derivation group and verify the relation between the predicted and measured ${\dot{\text{V}}\text{O}}_{2\max }$. When the absolute value of r falls within 0.00–0.10, 0.10–0.39, 0.40–0.69, 0.70–0.89, and 0.90–1.00, it corresponded to a negligible, weak, moderate, strong, and very strong relation, respectively^28^. Based on the participants’ EF6 or EF7 values during the CPET and other characteristic parameters, such as age, weight, BMI and PBF, this study established four ${\dot{\text{V}}\text{O}}_{2\max }$ prediction models through multiple linear regression analysis (randomly selecting 70% of the samples using SPSS statistical software [27.0, IBM Corp., USA] to establish the predictive model and utilizing the remaining 30% of the samples to validate the regression model). The BMI model included age and BMI. The PBF model^HRR^ included weight, HRR, and PBF. The PBF model^EF6^ incorporated weight, HRR, PBF, and EF6. The PBF model^EF7^ involved weight, HRR, PBF, and EF7. The goodness of fit and accuracy of these four prediction models were evaluated using multiple coefficients of determination (R^2^), the SEE, and the %SEE. This study adopted the PRESS statistical method to cross-validate these ${\dot{\text{V}}\text{O}}_{2\max }$ prediction models^11^. A Bland‒Altman plot was used to compare the difference between the measured and predicted ${\dot{\text{V}}\text{O}}_{2\max }$ in the derivation group^29^, and the mean difference ± 1.96 SD between the measured and predicted values was used to calculate the 95% limit of agreement (LoA). All data in this study were statistically analysed using SPSS, and descriptive data are presented as the means ± SDs. Statistical significance was considered at *p* < 0.05. With the use of G*Power software (version 3.1.9.7, Universität Kiel, Kiel, Germany), we computed a statistical power of 0.94 based on our sample size and an alpha level of 0.05.

## Results

The study results indicate that there were no significant differences between the derivation and validation groups in terms of age (20 ± 2 years vs. 21 ± 2 years), height (175.1 ± 6.0 cm vs. 174.8 ± 5.9 cm), weight (73.7 ± 10.6 kg vs. 73.3 ± 11.0 kg), BMI (24.0 ± 2.9 kg m^−2^ vs. 24.0 ± 3.3 kg m^−2^), PBF (16.9 ± 5.5% vs. 17.1 ± 6.3%), and HRR (128 ± 13 beats min^−1^ vs. 126 ± 12 beats min^−1^). Table 1 presents the important variables in the incremental test, including ${\dot{\text{V}}\text{O}}_{2\max }$, maximal workload, maximal heart rate, the RPE, and EFs.

**Table 1:** Descriptive physiological values of the incremental test and efficiency factors in models.

	Overall	Derivation group	Validation group	*p*
*N*	150	105	45	
${\dot{\text{V}}\text{O}}_{2\max }$ (mL kg^−1^ min^−1^)	39.0 ± 7.1	39.0 ± 7.0	38.9 ± 7.4	0.922^†^
Maximal workload (W)	182 ± 29	181 ± 28	184 ± 32	0.934^‡^
Measured maximal heart rate (beats min^−1^)	186 ± 6	186 ± 6	186 ± 5	0.881^‡^
Estimated maximal heart rate (beats min^−1^)	200 ± 2^#^	200 ± 2^#^	199 ± 2^#^	0.978^‡^
RPE	19 ± 1	19 ± 1	19 ± 1	0.833^‡^
EF1	1.41 ± 0.54	1.41 ± 0.57	1.40 ± 0.47	0.662^‡^
EF2	2.10 ± 0.85	2.07 ± 0.78	2.15 ± 1.00	0.804^‡^
EF3	2.25 ± 0.65	2.24 ± 0.63	2.28 ± 0.71	0.920^‡^
EF4	2.34 ± 0.62	2.34 ± 0.63	2.36 ± 0.63	0.969^‡^
EF5	2.39 ± 0.59	2.38 ± 0.60	2.41 ± 0.57	0.720^†^
EF6	2.39 ± 0.57	2.37 ± 0.58	2.44 ± 0.56	0.578^‡^
EF7	2.39 ± 0.55	2.37 ± 0.55	2.43 ± 0.53	0.621^‡^

${\dot{\text{V}}\text{O}}_{2\max }$ and RPE stand for maximal oxygen uptake and the rating of perceived exertion, respectively. EF1, EF2, EF3, EF4, EF5, EF6 and EF7 represent different levels of efficiency factors from stages 1 to 7 during the CPET. ^†^Refers to the *p* value of the independent samples t test for the derivation and validation groups. ^‡^Refers to the *p* value of the Mann‒Whitney *U* test for the derivation and validation groups. ^#^Refers to the significant difference between the estimated (220—age) and measured maximal heart rate, *p* < 0.001.

**Figure 1:**
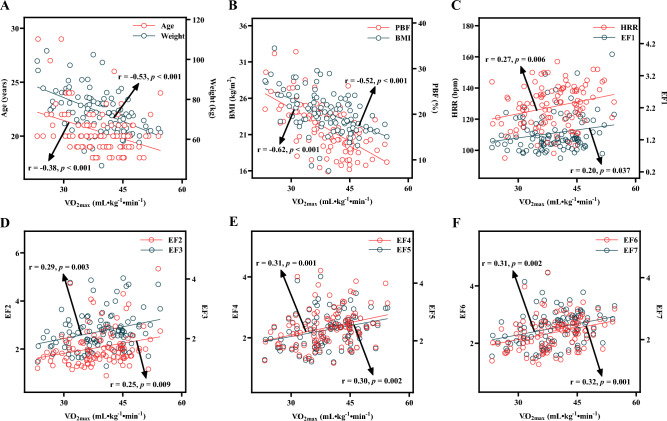
Scatter plot of independent variables of the derivative group and the measured ${\dot{\text{V}}\text{O}}_{2\max }$ (n = 105). BMI, PBF, HRR, and ${\dot{\text{V}}\text{O}}_{2\max }$ represent body mass index, percent body fat, heart rate reserve, and maximal oxygen uptake, respectively. EF1, EF2, EF3, EF4, EF5, EF6 and EF7 represent different levels of efficiency factors from stages 1 to 7 during the CPET.

Figure 1 presents the correlation coefficients between the measured ${\dot{\text{V}}\text{O}}_{2\max }$ and each independent variable. The results revealed a significant negative relation between ${\dot{\text{V}}\text{O}}_{2\max }$ and the variables of age (r = − 0.38, *p* < 0.001), weight (r = − 0.53, *p* < 0.001), BMI (r = − 0.52, *p* < 0.001), and PBF (r = − 0.62, *p* < 0.001), whereas the variables of HRR (r = 0.27, *p* = 0.006), EF1 (r = 0.20,* p* = 0.037), EF2 (r = 0.25,* p* = 0.009), EF3 (r = 0.29,* p* = 0.003), EF4 (r = 0.31,* p* = 0.001), EF5 (r = 0.30,* p* = 0.002), EF6 (r = 0.32,* p* = 0.001), and EF7 (r = 0.31,* p* = 0.002) were all significantly and positively related with ${\dot{\text{V}}\text{O}}_{2\max }$.

**Figure 2:**
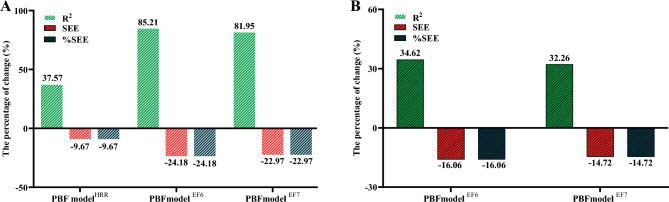
(**A**) Compared to that of the BMI model, the percentage change of PBF model^HRR^, PBF model^EF6^, and PBF model^EF7^ in R^2^, SEE, and %SEE; (B) Compared to that of the PBF model^HRR^, the percentage change of the PBF model^EF6^ and PBF model^EF7^ in R^2^, SEE, and %SEE. *PBF* percent body fat, *HRR* heart rate reserve, *EF6* efficiency factor of stage 6 during the CPET, *EF7* efficiency factor of stage 7 during the CPET, *R*^*2*^ multiple coefficients of determination, *SEE* standard error of estimate, *%SEE* SEE/mean of measured ${\dot{\text{V}}\text{O}}_{2\max }$ × 100.

Table 2 presents the four multiple regression models for predicting ${\dot{\text{V}}\text{O}}_{2\max }$, namely, the BMI model, PBF model^HRR^, PBF model^EF6^, and PBF model^EF7^. Figure 2 shows the percentage change in the prediction models for R^2^, SEE, and %SEE by comparing them to the BMI model (Fig. 2A) and PBF model^HRR^ (Fig. 2B). The study results indicated that, compared to that of the BMI model (R^2^ = 0.34, SEE = 5.8 mL kg^−1^ min^−1^, %SEE = 14.83%), the coefficient of determination (R^2^) of the PBF model^HRR^ (R^2^ = 0.47) increased by 37.57%, with a decrease of 9.67% in the error (SEE = 5.2 mL kg^−1^ min^−1^, %SEE = 13.39%); the R^2^ of the PBF model^EF6^ (R^2^ = 0.63) increased by 85.21%, with a decrease of 24.18% in the error (SEE = 4.4 mL kg^−1^ min^−1^, %SEE = 11.25%); and the R^2^ of the PBF model^EF7^ (R^2^ = 0.62) increased by 81.95%, with a decrease of 22.97% in the error (SEE = 4.5 mL kg^−1^ min^−1^, %SEE = 11.43%). Compared to that of the PBF model^HRR^, the R^2^ of the PBF model^EF6^ increased by 34.62%, with a decrease of 16.06% in the SEE or %SEE; the R^2^ of the PBF model^EF7^ increased by 32.26%, with a decrease of 14.72% in the SEE or %SEE (Fig. 2B). The PRESS cross-validation results indicated that the BMI model, PBF model^HRR^, PBF model^EF6^, and PBF model^EF7^ all had high stability (∆R^2^ < 0.01, ∆SEE < 0.3 mL kg^−1^ min^−1^; Table 2).

**Table 2:** Multiple regression model for the prediction of ${\dot{\text{V}}\text{O}}_{2\max }$ (mL kg^−1^ min^−1^).

	Variables	B	β	*p*	R^2^	SEE	%SEE	R^2^_*p*_	SEE_*p*_
BMI model	Constant	84.180		< 0.001	0.34	5.8	14.83	0.34	5.9
	Age (years)	− 0.891	− 0.268	0.002					
	BMI (kg/m^2^)	− 1.122	− 0.455	< 0.001					
PBF model^HRR^	Constant	48.116		< 0.001	0.47	5.2	13.39	0.47	5.3
	HRR (beats min^−1^)	0.105	0.200	0.008					
	Weight (kg)	− 0.176	− 0.264	0.004					
	PBF (%)	− 0.565	− 0.437	< 0.001					
PBF model^EF6^	Constant	47.856		< 0.001	0.63	4.4	11.25	0.62	4.7
	HRR (beats min^−1^)	0.080	0.152	0.017					
	Weight (kg)	− 0.390	− 0.586	< 0.001					
	PBF (%)	− 0.250	− 0.194	0.026					
	EF6	5.877	0.485	< 0.001					
PBF model^EF7^	Constant	47.833		< 0.001	0.62	4.5	11.43	0.61	4.7
	HRR (beats min^−1^)	0.078	0.149	0.021					
	Weight (kg)	− 0.399	− 0.599	< 0.001					
	PBF (%)	− 0.233	− 0.180	0.044					
	EF7	6.107	0.480	< 0.001					

*BMI* body mass index, *PBF* percent body fat, *HRR* heart rate reserve, ${\dot{\text{V}}\text{O}}_{2\max }$ maximal oxygen uptake, *EF6* efficiency factor of stage 6 during the CPET, *EF7* efficiency factor of stage 7 during the CPET, *B* unstandardized regression weights, *β* standardized regression weights, R^2^_*p*_ PRESS squared multiple correlation coefficient, *SEE* standard error of estimate, *SEE%* SEE/mean of measured ${\dot{\text{V}}\text{O}}_{2\max }$ × 100, *SEE*_*p*_ PRESS SEE.

**Figure 3:**
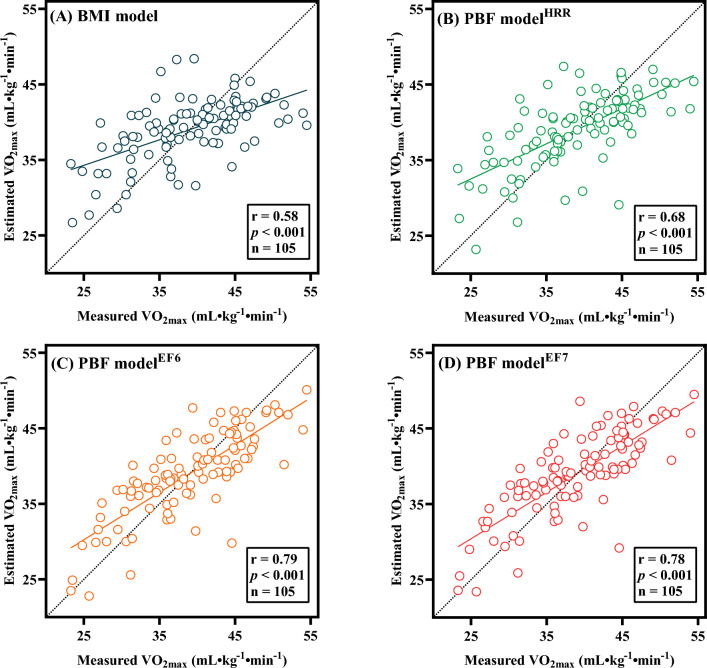
The association between the measured and predicted ${\dot{\text{V}}\text{O}}_{2\max }$: (**A**) BMI model; (**B**) PBF model^HRR^ model; (**C**) PBF model^EF6^; and (**D**) PBF model^EF7^. BMI, body mass index; PBF, percent body fat; HRR, heart rate reserve; EF6, efficiency factor of stage 6 during the CPET; EF7, efficiency factor of stage 7 during the CPET; ${\dot{\text{V}}\text{O}}_{2\max }$ maximal oxygen uptake.

Figure 3 shows the association between the measured and predicted ${\dot{\text{V}}\text{O}}_{2\max }$ in the derivation group. The statistical results indicated that the ${\dot{\text{V}}\text{O}}_{2\max }$ predicted by the BMI model (r = 0.58, *p* < 0.001), PBF model^HRR^ (r = 0.68, *p* < 0.001), PBF model^EF6^ (r = 0.79, *p* < 0.001), and PBF model^EF7^ (r = 0.78, *p* < 0.001) had a moderate to high relation with the measured ${\dot{\text{V}}\text{O}}_{2\max }$; among the models, the PBF model^EF6^ had the highest validity.

**Figure 4:**
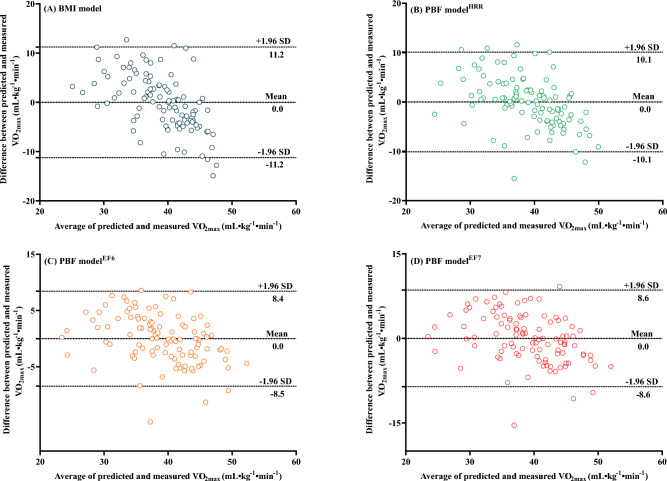
The difference between the measured ${\dot{\text{V}}\text{O}}_{2\max }$ and ${\dot{\text{V}}\text{O}}_{2\max }$ predicted by the BMI model (**A**), PBF model^HRR^ model (**B**), PBF model^EF6^ (**C**), and PBF model^EF7^ (**D**) in Bland‒Altman plots. *BMI* body mass index, *PBF* percent body fat, *HRR* heart rate reserve, *EF6* efficiency factor of stage 6 during the CPET, *EF7* efficiency factor of stage 7 during the CPET, ${\dot{\text{V}}\text{O}}_{2\max }$ maximal oxygen uptake.

The Bland‒Altman plots in Fig. 4 illustrate the difference between the measured and predicted ${\dot{\text{V}}\text{O}}_{2\max }$. The study results revealed that there was no significant difference between ${\dot{\text{V}}\text{O}}_{2\max }$ predicted by the BMI model (39.0 ± 4.1 mL kg^−1^ min^−1^), PBF model^HRR^ (39.0 ± 4.8 mL kg^−1^ min^−1^), PBF model^EF6^ (39.0 ± 5.6 mL kg^−1^ min^−1^), and PBF model^EF7^ (39.0 ± 5.5 mL kg^−1^ min^−1^) and the measured ${\dot{\text{V}}\text{O}}_{2\max }$ (39.0 ± 7.0 mL kg^−1^ min^−1^), with 95% LoAs of − 11.2 to 11.2 mL kg^−1^ min^−1^, − 10.1 to 10.1 mL kg^−1^ min^−1^, − 8.5 to 8.4 mL kg^−1^ min^−1^, and − 8.6 to 8.6 mL kg^−1^ min^−1^, respectively.

## Discussion

To meet the individual needs of healthy men, this study used predictive variables such as age, weight, BMI, PBF, HRR, and EFs to develop four ${\dot{\text{V}}\text{O}}_{2\max }$ prediction models: the BMI model, PBF model^HRR^, PBF model^EF6^, and PBF model^EF7^. The results of this study confirmed our hypothesis that there is indeed a significant positive relation between the EF and ${\dot{\text{V}}\text{O}}_{2\max }$ and that the PBF model^EF6^ established based on EF6 during the CPET has the highest accuracy, making it the best prediction model in this study. However, as the BMI model and PBF model^HRR^ are more economical and affordable, it is suggested that individuals can, according to their material conditions, select an appropriate prediction model for assessing or tracking their cardiorespiratory fitness.

In this study, demographic parameters such as age and weight were found to have a significant negative relation with ${\dot{\text{V}}\text{O}}_{2\max }$ (Fig. 1A), indicating a consistent result with previous studies on the prediction of ${\dot{\text{V}}\text{O}}_{2\max }$^30,31^. Many studies have found that age and weight are important variables for predicting ${\dot{\text{V}}\text{O}}_{2\max }$ in a cycle ergometer test^19,30–32^. The age-related ${\dot{\text{V}}\text{O}}_{2\max }$ levels can be classified into six categories: very poor, poor, fair, good, excellent, and superior^33^. On the other hand, oxygen uptake at a given load is closely related to the weight of participants. ${\dot{\text{V}}\text{O}}_{2\max }$ expressed in relative units (mL kg^−1^ min^−1^) is negatively related with weight^20^, whereas ${\dot{\text{V}}\text{O}}_{2\max }$ expressed in absolute units (L min^−1^) is positively related with weight^34^. Adding the weight parameter to regression models can enhance the power of predicting ${\dot{\text{V}}\text{O}}_{2\max }$^31,35^.

Previous studies have shown significant differences in cardiorespiratory fitness among adults with different BMI levels, with a higher BMI being associated with lower cardiorespiratory fitness^12,36^; overweight or obesity may increase the risk of cardiovascular disease in the general population^37,38^; and BMI and PBF are both important factors for the prediction of ${\dot{\text{V}}\text{O}}_{2\max }$, with PBF being a more accurate predictor of ${\dot{\text{V}}\text{O}}_{2\max }$ than BMI^11,16,27,39,40^. All of these findings were confirmed by the results of this study. In this study, BMI (r = − 0.52, *p* < 0.001) and PBF (r = − 0.62, *p* < 0.001) both had a significant negative relation with ${\dot{\text{V}}\text{O}}_{2\max }$. Nevertheless, compared to BMI, PBF had a stronger relationship with ${\dot{\text{V}}\text{O}}_{2\max }$ (Fig. 1B). Therefore, this study used BMI and PBF as independent predictors of ${\dot{\text{V}}\text{O}}_{2\max }$, based on which four ${\dot{\text{V}}\text{O}}_{2\max }$ prediction models—the BMI model, PBF model^HRR^, PBF model^EF6^, and PBF model^EF7^—were established.

Heart rate is an important physiological indicator that reflects the functions of the heart and circulatory system. Continuous heart rate measurement can directly reflect differences between individuals in terms of cardiac function status and sympathetic nervous system tension^41^, as well as their ability and adaptability to perform incremental exercise loads. In general, the human heart rate increases following an increase in exercise amount or intensity^42^. Individuals with different cardiorespiratory fitness can have different physiological responses under the same exercise load^42^. During the standard CPET process, the maximum heart rate of nonathletes usually approaches the maximum value of the age-predicted heart rate^43^. Individuals with higher cardiorespiratory fitness tend to have lower resting heart rates and exercise heart rates at the same work rate step^42,44^. The above viewpoints can be used to explain why there was a significant positive relation between HRR and ${\dot{\text{V}}\text{O}}_{2\max }$, as well as between various EFs (EF1, EF2, EF3, EF4, EF5, EF6, and EF7) and ${\dot{\text{V}}\text{O}}_{2\max }$ (Fig. 1C–F) in this study. Under fixed loads, individuals with higher cardiorespiratory fitness tend to have lower exercise intensity responses (%HRR), indicating a higher EF. Considering that all participants managed to complete the first seven stages of the CPET and that more than 2% of the participants failed to pass stage 8 and a higher resistance test, this study analysed only the EFs of the first seven CPET stages and used these EFs to derive the prediction models for ${\dot{\text{V}}\text{O}}_{2\max }$. By assessing participants’ exercise intensity responses and EFs during the CPET, it was possible to objectively understand their physical loads and improve the predictive power for ${\dot{\text{V}}\text{O}}_{2\max }$.

To meet the individual needs of different groups and determine the optimal prediction model, this study established the BMI model, PBF model^HRR^, PBF model^EF6^, and PBF model^EF7^ based on the linear relationship between ${\dot{\text{V}}\text{O}}_{2\max }$ and the variables of age, weight, BMI, PBF, HRR, and EFs. Among these models, the BMI model established based on age and BMI was the simplest and most economical model. Compared to the BMI model, the PBF model^HRR^ established based on HRR, weight, and HRR parameters had an R^2^ that increased by 37.57%, while its SEE or %SEE decreased by 9.67%. To further enhance the accuracy of predicting ${\dot{\text{V}}\text{O}}_{2\max }$, this study used EF6 and EF7, which were generated from the participants during the CPET, as the predictive factors to establish the PBF model^EF6^ and PBF model^EF7^, respectively. When the variable EF6 was added to the PBF model^HRR^, the R^2^ of the PBF model^EF6^ for ${\dot{\text{V}}\text{O}}_{2\max }$ increased by 85.21% and 34.62% compared to the BMI model and PBF model^HRR^, with decreases of 24.18% and 16.06% in the error, respectively. When the variable EF7 was added to the PBF model^HRR^, the R^2^ of the PBF model^EF6^ for ${\dot{\text{V}}\text{O}}_{2\max }$ increased by 81.95% and 32.26% compared to the BMI model and PBF model^HRR^, with decreases of 22.97% and 14.72% in the error, respectively. These results indicated that adding EF6 or EF7 to the models established based on biological data could significantly enhance the accuracy of the predicted ${\dot{\text{V}}\text{O}}_{2\max }$. Moreover, the PBF model^EF6^ had higher predictive accuracy for ${\dot{\text{V}}\text{O}}_{2\max }$ than the PBF model^EF7^, making it the best prediction model in this study. Therefore, instead of using the expensive ${\dot{\text{V}}\text{O}}_{2}$ analyser, the general public can accurately estimate their own ${\dot{\text{V}}\text{O}}_{2\max }$ using an adjustable resistance cycle ergometer, body composition scale and heart rate sensor. Currently, these devices are quite common and easily accessible in the market. Individuals who can afford these devices can consider estimating their ${\dot{\text{V}}\text{O}}_{2\max }$ based on the PBF model^EF6^, and for those with limiting conditions, the economical and budget-friendly BMI model or PBF model^HRR^ may be adopted as an alternative.

Many previous studies have successfully established ${\dot{\text{V}}\text{O}}_{2\max }$ prediction models using the submaximal cycling test^17,19,20,45^. The ${\dot{\text{V}}\text{O}}_{2\max }$ prediction formula established by Björkman et al.^18^ using the Åstrand submaximal cycling test can explain 50% of the variation in ${\dot{\text{V}}\text{O}}_{2\max }$, with an SEE of 5.6 mL kg^−1^ min^−1^. The validity correlation coefficient of ${\dot{\text{V}}\text{O}}_{2\max }$ predicted by Väisänen et al.^21^, Ekblom‐Bak et al.^23^, and Swain et al.^46^ using the Åstrand submaximal cycling test was 0.49–0.83, with SEEs of 5.8 mL kg^−1^ min^−1^, 0.5 L min^−1^, and 5.4 mL kg^−1^ min^−1^, respectively. Beekley et al.^22^ found a moderate relation (r = 0.63, *p* < 0.05) between the ${\dot{\text{V}}\text{O}}_{2\max }$ predicted through the YMCA submaximal cycling test and the measured ${\dot{\text{V}}\text{O}}_{2\max }$, with an SEE of 9.8 mL kg^−1^ min^−1^. Jamnick et al.^45^ established ${\dot{\text{V}}\text{O}}_{2\max }$ prediction formulas based on the YMCA and Mankato submaximal cycling tests, with r values of 0.64 and 0.72 and SEE values of 6.2 and 6.9 mL kg^−1^ min^−1^, respectively. In this study, the ${\dot{\text{V}}\text{O}}_{2\max }$ predicted by the BMI model, PBF model^HRR^, PBF model^EF6^, and PBF model^EF7^ had a medium to high relation with the measured ${\dot{\text{V}}\text{O}}_{2\max }$ (Fig. 3). Compared to previous reports on the prediction of ${\dot{\text{V}}\text{O}}_{2\max }$ through submaximal cycling tests, the prediction models established in this study are feasible.

Many previous studies on ${\dot{\text{V}}\text{O}}_{2\max }$ prediction often used the PRESS statistical method to cross-validate regression models and analyse the difference between the measured and predicted ${\dot{\text{V}}\text{O}}_{2\max }$ in Bland‒Altman plots^11,12,27,47^. To further evaluate the effectiveness of the BMI model, PBF model^HRR^, PBF model^EF6^, and PBF model^EF7^, this study also adopted PRESS and Bland‒Altman plots to validate these models. The results of the PRESS statistical analysis indicated that the BMI model, PBF model^HRR^, PBF model^EF6^, and PBF model^EF7^ all had a high level of cross-validity (∆R^2^ < 0.01; Table 2). There were no significant differences between the measured ${\dot{\text{V}}\text{O}}_{2\max }$ and ${\dot{\text{V}}\text{O}}_{2\max }$ estimated by the BMI model, PBF model^HRR^, PBF model^EF6^, and PBF model^EF7^ (Fig. 4). Among the models, the consistency between the measured ${\dot{\text{V}}\text{O}}_{2\max }$ and that predicted by the PBF model^EF6^ was the highest_._ The above results indicate that EFs during the CPET are effective predictors of ${\dot{\text{V}}\text{O}}_{2\max }$, with EF6 being the optimal predictor. Therefore, the ${\dot{\text{V}}\text{O}}_{2\max }$ prediction model established using EF6 is more accurate and reasonable.

## Limitations

This study has some limitations. First, all participants were healthy males aged 18–30 years. Therefore, it is not possible to verify whether the study results are applicable to females and males aged above 30 years old. Additionally, it is not suitable for individuals who taking medications, such as betablockers, as our model relies on HRR. Second, the CPET was carried out on an electrically braked cycle ergometer instead of a treadmill or other sports equipment, and different exercise models can result in a difference in measured ${\dot{\text{V}}\text{O}}_{2\max }$. Third, this was a cross-sectional study, and a longitudinal study design may provide a more comprehensive analysis for the development of ${\dot{\text{V}}\text{O}}_{2\max }$ prediction models. Finally, there is a difference between estimated (220-age) and measured maximum heart rate, leading to a discrepancy in estimated and measured HRR, thereby potentially affecting the accuracy of ${\dot{\text{V}}\text{O}}_{2\max }$ prediction.

## Conclusions

This study has proven the significant positive relation between the EF and ${\dot{\text{V}}\text{O}}_{2\max }$. Multiple regression models established based on EF6/EF7 can effectively enhance the accuracy in predicting ${\dot{\text{V}}\text{O}}_{2\max }$. Nevertheless, considering the differences among the general population in terms of material conditions and individual needs, this study established four prediction models, including the BMI model, PBF model^HRR^, PBF model^EF6^, and PBF model^EF7^. Among these models, the PBF model^EF6^ established based on HRR, weight, PBF, and EF6 is considered the best prediction model due to it showing the highest accuracy.

## Data Availability

The data that support the findings of this study are available upon request from the corresponding author.
